# Eltrombopag and its iron chelating properties in pediatric acute myeloid leukemia

**DOI:** 10.18632/oncotarget.28000

**Published:** 2021-07-06

**Authors:** Maura Argenziano, Chiara Tortora, Alessandra Di Paola, Elvira Pota, Martina Di Martino, Daniela Di Pinto, Caterina Di Leva, Francesca Rossi

**Affiliations:** ^1^Department of Woman, Child and General and Specialist Surgery, University of Campania Luigi Vanvitelli, Naples 80138, Italy; ^2^Department of Experimental Medicine, University of Campania Luigi Vanvitelli, Naples 80138, Italy

**Keywords:** acute monocytic leukemia, eltrombopag, deferasirox, iron chelation, cancer

## Abstract

Pediatric acute myeloid leukemia (AML) represents 20% of total childhood leukemia diagnoses and is characterized by poor prognosis with a long-term survival rate around the 50%, when patients are properly treated. The standard treatment for pediatric AML currently consists in a combination of cytarabine (Ara-C) and antracycline. Iron plays an important role in cancer development and progression. Targeting iron and its metabolism mediators could be a novel therapeutic strategy in cancer.Deferasirox (DFX) inhibits cancer cell proliferation and its use as an antiblastic drug could be suggested. Eltrombopag (ELT), a thrombopoietin receptor agonist used in immunethrombocytopenia, shows anticancer properties related to its emerging iron chelating properties. We compare the anticancer effect of classically used cytarabine with DFX and ELT effects in a pediatric AML cell line, THP-1, in order to identify innovative and more effective therapeutic strategies. ELT and DFX reduce intracellular iron concentration by inhibiting its uptake and by promoting its release. In particular, even though further investigations are needed to better understand the extact underlying action mechanisms, we demonstrated that ELT improves cytarabine antineoplastic activity in pediatric AML cell line.

## INTRODUCTION

Acute myeloid leukemia (AML) represents the 20% of total childhood leukemia diagnoses [1], even though it remains the most frequent type of acute leukemia in the elderly [2]. In 0–2 year-old children, this kind of leukemia is characterized by poor prognosis, with a survival rate around the 60% [3], and high treatment-related toxicity [4]. In infants the most common subtype of AML is the acute monocytic leukemia (AMoL) responsible for high mortality and morbidity [5].

The combined administration of cytarabine (Ara-C) and antracycline currently represents the principal therapeutic strategy for AML in pediatric patients, while for post-remission therapy repeated courses of high-dose Ara-C together with other cytotoxic agents are strongly indicated [3]. Also allogeneic hematopoietic stem cell transplantation (HSCT) is widely utilized to consolidate the state of remission [6], even though its actual benefits in comparison to conventionally used chemotherapy has been questioned in last years since the observed risk of relapse and late side effects. Although the amelioration of outcome for this pathology [7], stronger efforts to overcome the side effects related to standard therapy and also to ameliorate the life quality of patients are needed. Nowadays the involvement of iron and its metabolism mediators in tumor onset and progression is well documented, including in AML [8]. Considering the importance of iron in both physiological and pathological cell functions, any alterations in its metabolism could be very deleterious for human health. In particular, in literature it is reported a strong correlation between iron excess and cancer onset and progression [9], since the great request showed from cancer cells to sustain their metabolic processes. In AML iron overload is very frequent and could be defined as “primary”, due to deficit in erythropoiesis, or “secondary”, related to the repeated red blood cells transfusions principally aimed to counteract anemia [10]. Anyway, this condition has been seen to worsen AML symptoms, contributing to bone marrow failure [11] and to immune response decrease [12]. Therefore, serum and cellular iron levels are valid prognostic factors in predicting patients’ response to therapy or also hematopoietic stem cell transplantation outcome [13, 14]. The altered iron metabolism in AML patients is also strongly related to a dysregulation in the expression of iron metabolism mediators at AML cells level. For example, these cells overexpress the transferrin receptor, TFRC, for the internalization of transferrin-iron complexes, thus demonstrating their high iron consumption [15–17]. On these bases, contrasting iron overload could represent an effective anticancer strategy also in AML [18, 19].

European Medicines Agency (EMA) approved three iron chelators for treatment of iron overload conditions: deferoxamine (DFO) generally used in thalassemia major, deferiprone (DFP) and deferasirox (DFX) instead indicated in leukemic patients. In particular, DFX has also anticancer properties since it limits cancer cell proliferation by inhibiting NF-kB [20] and enhances p53 transcriptional activity impairing leukemic cells growth [21, 22]. Besides these compounds, other molecules with iron chelating properties are under investigations and among them one of the most promising is Eltrombopag (ELT), a thrombopoietin receptor agonist, normally indicated for use in immunethombocytopenia (ITP) and aplastic anemia. Vlachodimitropoulou et al. demonstrated that ELT mobilizes iron from intracellular compartment, thus reducing the metal availability for cancer cell metabolic processes, and proposed a shuttling mechanism when ELT and DFX are combined. In particular, ELT decreases cellular iron and further enhances iron mobilization donating it to DFX. [23]. However, the possibility to use ELT in AML patients is controversial. In 2018 Mittelman et al. observed a lower rate of clinically relevant thrombocytopenic events in the group treated with ELT [24]. In contrast, Frey and collaborators revealed an increase in adverse events after the administration of ELT in patients [25].

In this study we tested cytarabine, the most commonly used anticancer drug in AML, with both DFX, one of the most diffused iron chelators in leukemia, and ELT, an emerging iron chelating agent, in THP-1 cell line. The purpose of our study was to investigate the possibility to combine iron chelation with standard anti-leukemic treatments, to ameliorate the therapeutic outcome for leukemic pediatric patients, contributing to improve therapy response and consequent life quality.

## RESULTS

### Effects of treatments on viability

We evaluated the effect of ELT, DFX and Cytarabine, alone and in combination, on THP-1 cells viability, after 48 h of exposure by means of a specific cytofluorimetric assay. At the chosen concentrations, ELT and DFX did not cause any alteration in cell viability, indicating they are not cytotoxic for THP-1 cells, differently from Cytarabine which, as expected being it a chemotherapy drug, caused a very strong reduction of viable cells. A similar reduction in viability is observed also after all the co-treatments. In particular, the cytotoxicity of Cytarabine is exacerbated when used in combination with ELT (Table 1).

**Table 1:** Cell viability evaluation after 48-hour treatments

	Cell Viability %
**NT**	91, 25 ± 5, 9
**ELT**	83, 10 ± 1, 7
**DFX**	90, 45 ± 8, 5
**CYT**	54, 40 ± 1, 2^*^
**ELT + DFX**	74, 40 ± 4, 1^*^
ELT + CYT^*^	44, 90 ± 6, 9^*^
DFX + CYT^*^	60, 30 ± 2, 5^*^

The table shows the percentage of cell viability after 48-hour treatment with ELT, DFX and Cytarabine, alone and in combination. The experiment was run in triplicate and results showed as mean ± SD. Student’s *t* test was used for statistical analysis. *p* ≤ 0.05 was considered statistically significant, ^*^ vs non-treated sample.

^*^ *p* ≤ 0,05.

### Effects of treatments on iron metabolism

**Figure 1:**
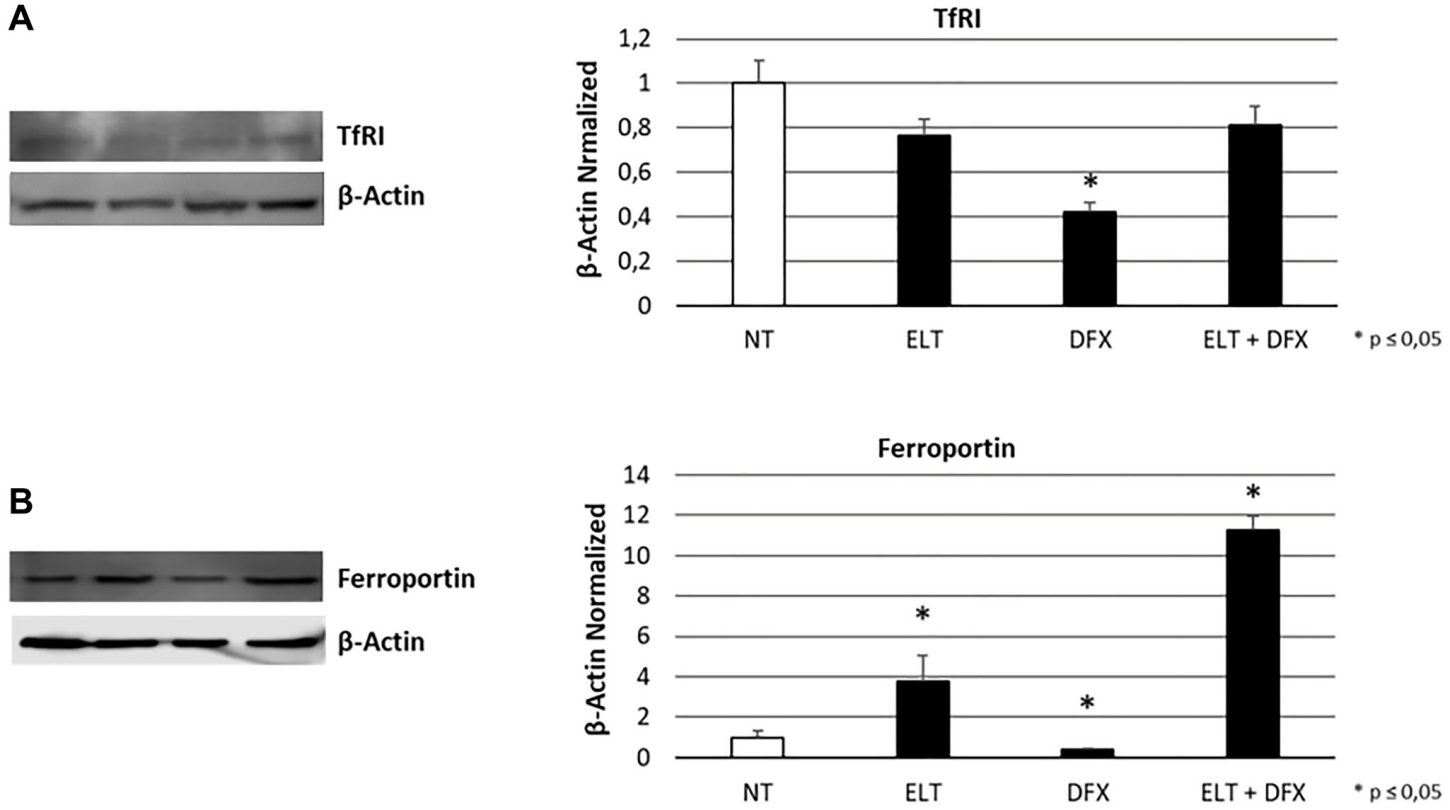
Effect of ELT and DFX on iron metabolism mediators. TfR1 (**A**) and Ferroportin (**B**) proteins density in THP-1 cell line was determined by Western blotting, starting from 15 μg of total lysates and after 48 h exposure to ELT and DFX, alone and in combination. The most representative cropped images of blots are displayed. The proteins were detected using Image Studio Digits software, and the intensity of immunoblots compared to the untreated control, taken as 1 (arbitrary unit), were quantified after normalizing with respective loading controls for the housekeeping protein β-Actin. Histograms show protein expression levels as the mean ± S.D. of three replicates. A *t*-test was used for statistical analysis. ^*^ indicates *p* ≤ 0.05 compared to non-treated sample (NT).

**Figure 2:**
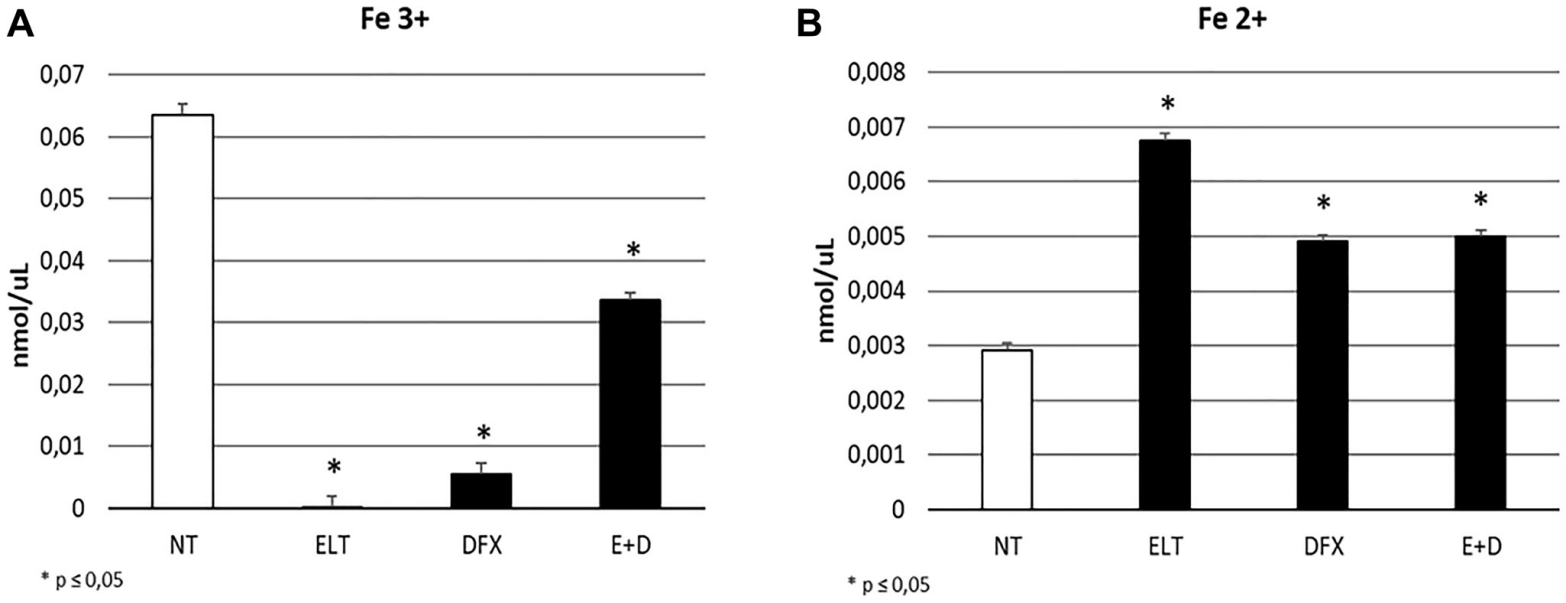
Effect of ELT and DFX on iron import and release. Fe3+ intracellular concentrations (nmol/μL) (**A**) and Fe 2+ extracellular concentrations (nmlo/μL) (**B**) in THP-1 cell were determined by Iron Assay, after treatment with ELT and DFX, alone and in combination. Histograms show Fe3+ and Fe2+ concentrations as mean ± S.D. of three independent replicate. A *t*-test was used for statistical analysis. ^*^ indicates *p* ≤ 0.05 compared to NT.

To evaluate the influence of ELT and DFX, alone and in combination, on iron metabolism of THP-1 cells, we performed Western Blotting analyzing the expression levels of two key modulators of iron metabolism: transferrin receptor 1 (TFR-1) and Ferroportin. TRF-1 is responsible for iron internalization and after 48-hour exposure to ELT and DFX its expression was reduced but only with DFX in a statistically significant manner (Figure 1A). Ferroportin is the only known iron exporter protein. We observed its decrease after DFX administration and its increase after ELT administration. This increase is even more marked when ELT and DFX are combined (Figure 1B). These results are in line with the colorimetric iron assay by means we observed a reduction of intracellular Fe^3+^ (ferric iron) (Figure 2A) and a contemporary increase of extracellular Fe^2+^ (ferrous iron) (Figure 2B), after all treatments but especially after ELT exposure.

### Effects of treatments on apoptosis

**Figure 3:**
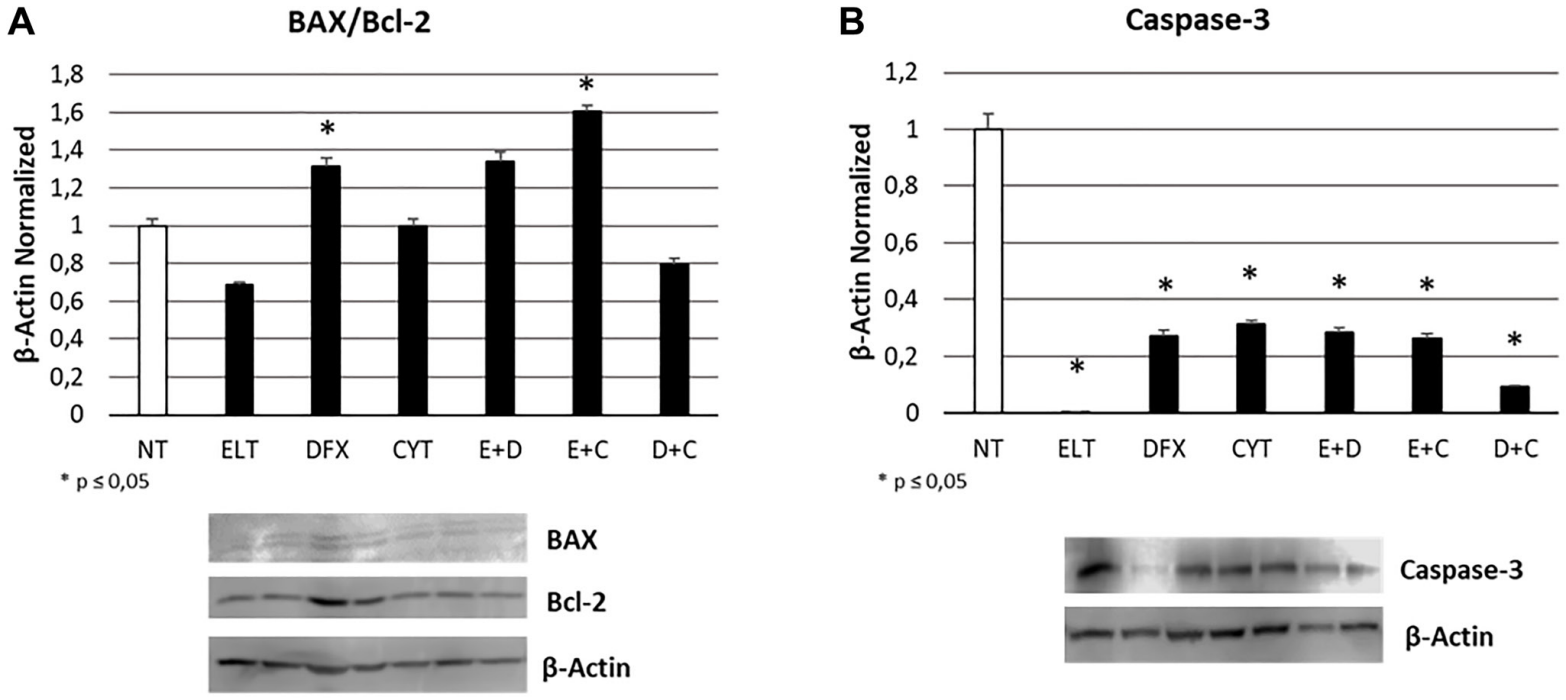
Effect of 48-hour treatments on apoptosis. BAX/Bcl-2 proteins ratio (**A**) and Caspase-3 protein (**B**) density in THP-1 cell line was determined by Western blotting, starting from 15 μg of total lysates and after 48 h exposure to ELT, DFX and Cytarabine, alone and in combination. The most representative cropped images of blots are displayed. The proteins were detected using Image Studio Digits software, and the intensity of immunoblots compared to the untreated control, taken as 1 (arbitrary unit), were quantified after normalizing with respective loading controls for the housekeeping protein β-Actin. Histograms show protein expression levels as the mean ± S.D. of three replicates. A *t*-test was used for statistical analysis. ^*^ indicates *p* ≤ 0.05 compared to non-treated sample (NT).

After 48-hour exposure of THP1 cell line to ELT [10 μM], DFX [10 μM] and Cytarabine [5 μM], alone and in combination, we studied the effects on apoptosis by means of a cytofluorometric assay on Muse Cell Analyzer (Merk-Millipore) and by Western Blotting evaluating BAX/Bcl-2 and Caspase-3 protein expression. In particular, BAX protein has a pro-apoptotic role, while Bcl-2 an anti-apoptotic function. Caspase- 3 is instead one of the main effector protein of apoptosis. The increase in BAX/Bcl-2 ratio as well as in Caspase-3 expression levels indicates an anti-tumoral effect of treatment. We observed a statistically significant increase in BAX/Bcl-2 ratio only after treatment with DFX alone and ELT plus Cytarabine (Figure 3A), on the contrary Caspase-3 decreased after all the administered treatments (Figure 3B). This results only in part resemble what observed with cytofluorimetric assay (Table 2), which highlighted a statistically significant increase in apoptotic cell percentage after treatments with ELT and Cytarabine alone and also after all the co-treatments, even though it is evident that the expected pro-apoptotic effect of the anticancer Cytarabine is ameliorated only by ELT.

**Table 2:** Apoptosis evaluation after 48-hour treatments

%	Total Apoptosis
**NT**	34, 23 ± 3, 6
**ELT**	45, 98 ± 9, 9
**DFX**	36, 85 ± 2, 5
**CYT**	66, 55 ± 5, 9^*^
**ELT + DFX**	61, 98 ± 2, 5
**ELT + CYT**	73, 50 ± 6, 7^*^
**DFX + CYT**	62, 25 ± 6, 6^*^

The table shows the percentage of total apoptosis after 48-hour treatment with ELT, DFX and Cytarabine, alone and in combination. The experiment was run in triplicate and results showed as mean ± SD. Student’s *t* test was used for statistical analysis. *p* ≤ 0.05 was considered statistically significant, ^*^ indicates *p* ≤ 0.05 compared to non-treated sample (NT).

^*^ *p* ≤ 0,05.

### Effects of treatments on cell cycle progression

**Figure 4:**
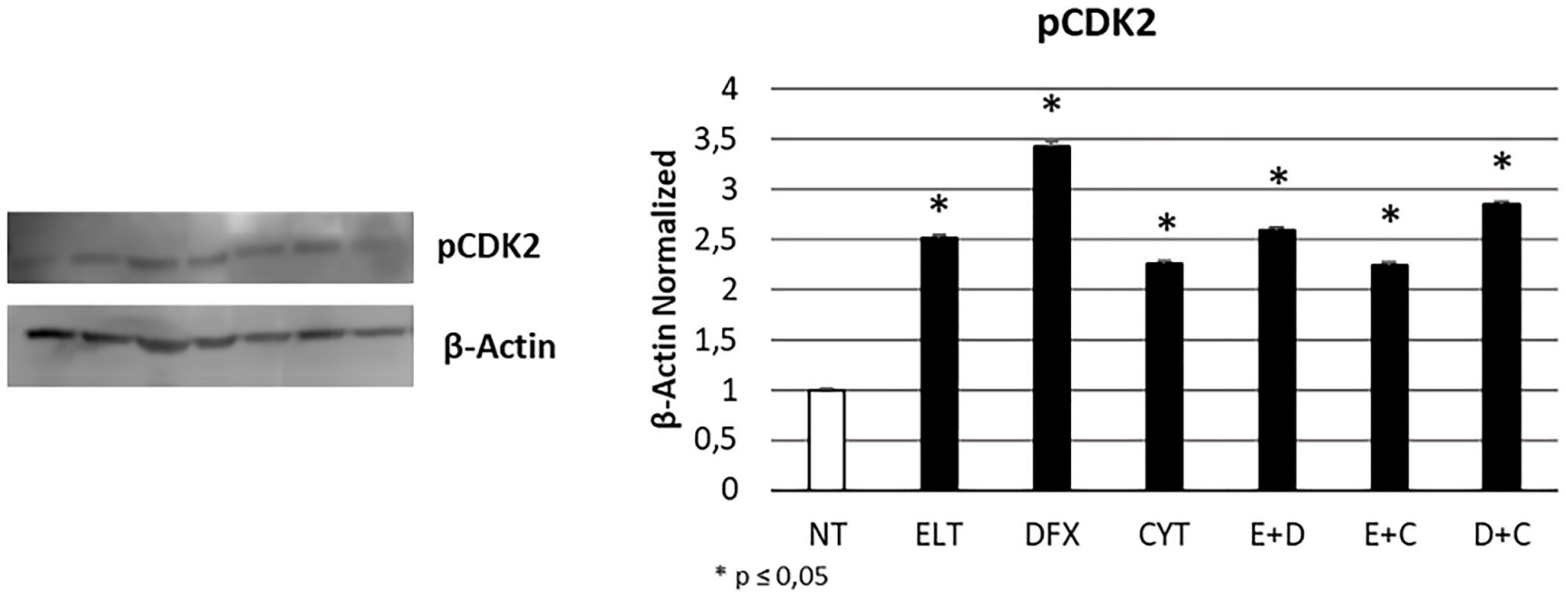
Effect of 48-hour treatments on cell cycle progression. pCDK2 protein expression levels in THP-1 cell line were determined by Western Blot, starting from 15 μg of total lysates after 48 h of exposure to ELT, DFX and Cytarabine, alone and in combination. The most representative cropped images of blots are displayed. The proteins were detected using Image Studio Digit software and the intensity ratios of immunoblots compared to that of untreated control, taken as 1 (arbitrary unit), were quantified after normalizing with respective loading controls for the housekeeping protein β-Actin. The histogram represents the relative quantification for pCDK2 expression as mean ± SD of three independent experiments A *t*-test has been used to evaluate the statistical differences in protein expression levels. ^*^ indicates *p* ≤ 0.05 compared to non-treated sample (NT).

To evaluate the effects of 48 hour-treatment with ELT, DFX and Cytarabine, alone and in combination, on cell cycle progression, we performed a specific cytofluorimetric assay on the Muse Cell Analyzer and also a Western Blotting for pCDK2 protein expression, a key kinase in mediating cell passage from G0/G1 to S phase. Western Blotting evidenced a significant increase of its expression after all treatments (Figure 4). While only after treatment with ELT and Cytarabine in combination we observed at Muse cell analyzer an accumulation of THP-1 cells in G0/G1 phase in comparison with non-treated sample (NT), maybe not related to pCDK2 protein activity (Table 3).

**Table 3:** Cell cycle evaluation after 48-hour treatments

%	G0/G1	S	G2/M
**NT**	47, 6 ± 2, 7	20, 5 ± 4, 8	28, 5 ± 4, 4
**ELT**	30, 3 ± 5, 1^*^	29, 5 ± 0, 4^*^	26 ± 2, 0
**DFX**	36, 75 ± 2, 0^*^	27, 3 ± 5, 5^*^	31 ± 4, 5^*^
**CYT**	32 ± 6, 5^*^	48, 7 ± 7, 4^*^	16, 1 ± 8, 6^*^
**ELT + DFX**	33, 9 ± 6, 5^*^	29, 1 ± 8, 1^*^	33, 3 ± 3, 4^*^
**ELT + CYT**	71, 9 ± 12, 6^*^	15, 6 ± 1, 2^*^	10, 7 ± 1, 5^*^
**DFX + CYT**	29, 5 ± 0, 8^*^	54, 2 ± 7, 9^*^	14, 1 ± 1, 7^*^

Percentage of THP-1 cell cells at different phases of the cell cycle after 48 h of exposure to ELT, DFX and Cytarabine, alone and in combination. Cells were harvested, fixed, incubated with Muse Cell Cycle reagents and analyzed on “MUSE Cell Analyzer”. The results are presented as the mean percentage ± SD of three independent experiments and were analyzed by *t*-test. ^*^ indicates *p* ≤ 0.05 compared to non-treated sample (NT).

^*^ *p* ≤ 0,05.

### Effects of treatments on proliferation

**Figure 5:**
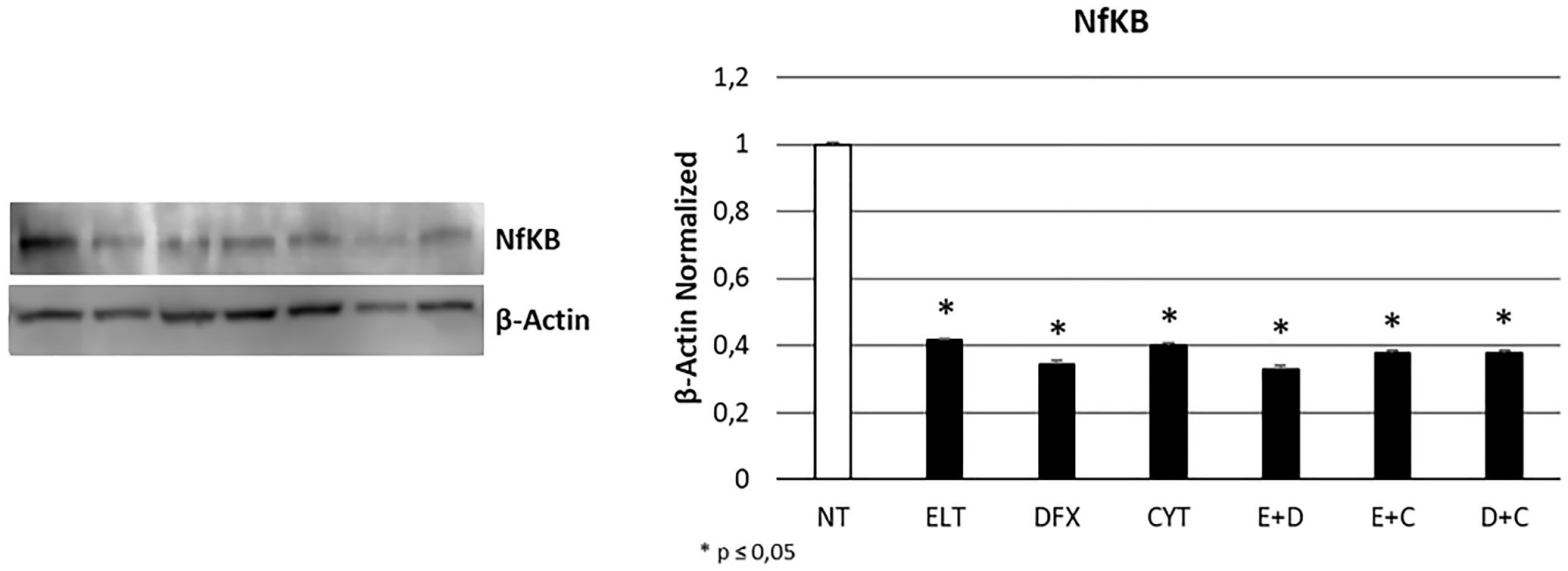
Effect of 48-hour treatments on proliferation. NFkB protein expression level in THP-1 cell line was determined by Western Blot, starting from 15 μg of total lysates after 48 h of exposure to ELT, DFX and Cytarabine, alone and in combination. The most representative cropped images of blots are displayed. The proteins were detected using Image Studio Digit software and the intensity ratios of immunoblots compared to that of untreated control, taken as 1 (arbitrary unit), were quantified after normalizing with respective loading controls for the housekeeping protein β-Actin. The histogram represents the relative quantification for NFkB expression as mean ± SD of three independent experiments A *t*-test has been used to evaluate the statistical differences in protein expression levels. ^*^ indicates *p* ≤ 0.05 compared to non-treated sample (NT).

To evaluate whether ELT, DFX and Cytarabine affect THP-1 cells proliferation, we performed Western Blotting for nuclear factor kappa-light-chain-enhancer of activated B cells protein (NF-kB), strongly involved in cancer progression. All treatments reduced its expression level to more than half with a statistically significant difference with the non-treated sample (NT) (Figure 5).

## DISCUSSION

Even though acute myeloid leukemia (AML) principally affects elderly [2], it represents the 20% of childhood leukemia cases [1]. The most relevant aspect of pediatric AML remains the poor prognosis [26] with a long-term survival rate around the 50% [27], when patients are properly treated. The induction therapy combining cytarabine (Ara-C) and antracycline is nowadays the most diffuse and effective therapeutic approach to treat AML pediatric patients [3]. Anyway, even though in the last years the outcome ameliorates for these patients, stronger efforts to better manage this pathology and to improve the life quality of children in long-term are needed.

In the last decade the role of iron in cancer onset and progression emerged [28, 29]. In particular, since tumor cells show higher iron consumption than healthy cells [30], iron chelating agents represent a promising anticancer strategy. Several authors demonstrated that iron chelators inhibit proliferation and induces apoptosis in both hematological and solid tumors [21, 31]. For example, the iron chelators deferiprone (DFP) and deferasirox (DFX) are two iron chelators approved for use in leukemic patients, being able to inhibit cancer cell proliferation [20–22]. Among the innovative molecules under investigations for their emerging iron chelating properties, eltrombopag (ELT), the thrombopoietin receptor agonist used in immunethrombocytopenia (ITP) and aplastic anemia, shows anticancer properties related to its iron chelating properties [23], even though the possibility of using it is not yet concrete [24, 25, 32].

We compared the anticancer effect in THP-1 cell line of the classical chemotherapy drug, cytarabine, of DFX, the iron chelator already used in leukemic patients, and ELT, an emerging iron chelator in anticancer therapy. Moreover, we combined them with each other to investigate the possibility of a synergism between iron chelation and classical chemotherapy, in order to improve patients’ response, as well as to reduce the resistance they often develop against cytarabine [33]. First of all, we confirmed the iron chelating properties of ELT and DFX also in THP-1 cell line. They indeed reduce the intracellular levels of Fe3+, thus making ferric iron less available for cellular vital functions. This result could be related to the observed reduction in TFR-1 expression level and to the contemporary increase in Ferroportin. These proteins are the two of the principal actors in iron metabolism: TFR-1 is responsible for iron intake and Ferroportin for iron release in the extracellular microenvironment. In literature the evidences about the role of Ferroportion in cancer are discordant. Indeed, while in the major part of cancers low levels of ferroportin are related to poor prognosis [34, 35], in 2019 Gasparetto et al. observed an improved outcome to chemotherapy in AML expressing lower levels of ferroportin [14]. This difference could be due to biological differences between cancers and also to differences in therapy strategies. Even though the role of ferroportin in cancer need to be better clarified, in literature it is clear the potential anticancer role of iron chelation and in particular ELT seems to be a very promising agent in this field [36, 37].

Hence, we studied the effect of treatments on cell viability, apoptosis, cell cycle progression and proliferation. First of all, we showed that cytarabine reduced cell viability and also increases apoptosis levels in THP-1 cells, as already demonstrated in other leukemia cell lines [38]. DFX and ELT instead did not affect cell viability when alone but exacerbated the effect of cytarabine, especially ELT, thus letting hypothesize a synergism between the classical chemotherapy and the innovative anticancer drug ELT. Indeed, after co-treatment with ELT and cytarabine, cell viability reduced of almost 50%, more than after each single treatment or co-treatment with DFX. ELT is able also to increase the pro-apoptotic effects of cytarabine, as demonstrated by cytofluorimetry and by the significant increase in BAX/Bcl-2 protein expression levels ratio. Bcl-2 protein is normally responsible for inhibiting apoptosis and in case of resistance to cytarabine it is often up-regulated [38]. An increase in BAX/Bcl-2 ratio indicates a prevalence of the pro-apoptotic processes and interestingly in THP-1 cell line this beneficial anticancer effect is evident only when cytarabine is combined with ELT. This result supports the anti-cancer effectiveness of ELT, but on the other hand it must to be better clarified considering also what reported in 2019 by Yao et al. They demonstrated how to antagonize iron-overload can instead promote Bcl-2 and inhibit BAX expression, limiting the pro-apoptotic processes in healthy BMSCs [39]. This controversial aspect on iron overload management could depend on the considered cell type and health condition. As regards cell cycle progression, we observed a block in S phase after cytarabine administration in THP-1 cells, as also reported for decitabine, another chemotherapy drug currently used in AML not eligible for standard protocols [37]. But, when we combined it with ELT, the arrest shifted in G0/G1 phase, consistently with the already documented effects of ELT used alone [37, 40]. Several authors demonstrated that iron chelators reduce cyclin-dependent kinase 2 (CDK2) activity [41–43], a protein normally responsible for cell cycle progression from G0/G1 phase to S one. Deregulation in its function or expression are associated to many human cancers [44], so much that CDK2 inhibition represents a very promising anti-tumor strategy. All treatments we administered caused a significative increase in CDK2 expression, which is not in accordance with the arrest actually observed by cytofluorimetry. Therefore, it could be interesting to investigate on the hypothesis formulated by Shi et al*.,* which observed an arrest in G0/G1 phase after treating leukemia cells with ELT and decitabine [37] and attributed this anticancer effect to the alteration in intracellular ROS levels caused by ELT [40]. We also evaluated the effect of treatments on cell proliferation by analyzing the expression levels of nuclear transcription factor-kappa beta (NFkB), one of the principal mediators of inflammation, carcinogenesis [45, 46] and chemotherapy resistance [38]. NFkB pathway is also involved in iron metabolism and in particular its inhibition reduces iron overload [47]. All treatments we administered in THP-1 cell line caused a significant reduction in its expression, thus mediating an important anti-proliferative effect and also a useful secondary effect of iron overload containment.

In conclusion, our study lets emerge a promising synergism between ELT and cytarabine with reduction in viability, increase in apoptosis and arrest of both proliferation and cycle progression in the pediatric AML cell line THP-1. On the other hand, we can not confirm the effectiveness of DFX in AML, neither alone neither in combination with the classically used chemotherapy agent. Further investigations are certainly needed to clarify the exact mechanisms underlying the synergism between ELT and cytarabine, in particular to understand whether ELT iron chelating properties are actually responsible for these anticancer activities. However our results encourage the possibility to combine them to increase the outcome of canonic therapeutic strategy in AML, reducing dose-related side effects associated to cytarabine as well as the chemo-resistance often developed by patients against this agent.

## MATERIALS AND METHODS

### Leukemic cell line

THP-1 cells are monocytes derived from 1-year-old infant affected by acute monocytic leukemia. This cell line was purchased from ATCC and cultured in suspension in RPMI-1640 Medium (Lonza, Verviers, Belgium), supplemented with 10% fetal bovine serum (FBS) (Euroclone, Siziano, Italy), 100 IU/mL penicillin, and 100 g/mL streptomycin and L-glutamine (Gibco Limited, Uxbridge, UK). Cells were cultured at 37°C in a humidified atmosphere with 5% CO_2_. 48 h after thawing out, cells were harvested, washed and counted on a microscope using a Burker Haemocytometer and 1,0 × 10^6^ cells per well were plated in a 6 multiwell. Once 80% confluence was reached, Eltrombopag [10 μM], Deferasirox [10 μM] and Cytarabine [5 μM] were added alone and in combination. Cells were harvested at 48 h for Western Blotting, Iron Assay and cytofluorimetric assays to evaluate viability, apoptosis and cell cycle progression.

### Drugs and treatments

ELT and DFX are two iron chelators purchased from Novartis S.p.a. (Origgio, VA, Italy). They were dissolved in sterile water at a concentration of 10 mM for ELT and 5 mM for DFX. Stock solutions were aliquoted and kept at −80°C for long-term storage. Cytarabine is an anti-cancer drug, belonging to the antimetabolites category. Its anti-cancer effects are due to the inhibition of DNA production and repair. It is indicated for treatment of leukemia, Hodgkin’s lymphoma and other types of lymphoma and it is commercialized as Cytosar-U^**®**^. We diluted the initial injectable solution of 100 mg/mL to use it at the final concentration of [5 μM] in each well. THP-1 cell line was treated with ELT at the final concentration of [10 μM], DFX at [10 μM] and Cytarabine at [5 μM], both alone and in all the possible combination. The used concentrations were determined following a pilot Dose-Response experiment (data not shown). Non-treated cultured cells were maintained in incubation media during the relative treatment time with and without a vehicle (sterile water).

### Protein isolation and western blot

Proteins were extracted from treated and non-treated THP-1 cells using radio-immunoprecipitation assay (RIPA) Lysis Buffer (Millipore, Burlington, MA, USA) following the were detected in total lysates from cell line cultures by Western Blotting. Membranes were incubated overnight at 4°C with these antibodies: goat polyclonal anti TfR1 (1:1000, Thermofisher), rabbit polyclonal anti ferroportin (1:1000, NOVUS), mouse monoclonal anti BAX (1:100, Santa Cruz), mouse monoclonal anti Bcl-2 (1:200, Santa Cruz), rabbit polyclonal anti Caspase 3 (1:2000, Bethyl Laboratories, Inc.), rabbit monoclonal anti pCDK2 (1:1000, abcam), rabbit polyclonal anti NFkB (1:500, Bethyl Laboratories, Inc.). Reactive bands were detected by chemiluminescence (Immobilon Western Chemiluminescent HRP Substrate, Millipore, Burlington, MA, USA) on a C-DiGit^®^ Blot Scanner (LI-COR Biotechnology^®^, Lincoln, NE, USA). A mouse monoclonal anti β-Actin antibody (1:100, Santa Cruz) was used to check for comparable protein loading and as a housekeeping protein. Images were captured, stored and analyzed using “Image studio Digits ver. 5.0” software.

### Iron assay

After 48 hour-treatment, cell culture supernatants were collected to measure iron (III) and iron (II). The assay was performed by using the Iron Assay Kit (Abcam, Cambridge, UK) according to the manufacturer’s instructions. Briefly, standards and THP-1 cells supernatants and lysates were pipetted into the wells and were incubated with an acidic buffer to allow iron release. Then, an iron probe at 25°C for 60 min was added, protected from light. Released iron reacted with the chromogen resulting in a colorimetric (593 nm) product, proportional to the iron amount. The optical density was measured at a wavelength of 593 nm by using the Tecan Infinite M200 (Tecan Group Ltd., Männedorf, Switzerland) spectrophotometer. Iron (II) and Total Iron (II + III) contents of the test samples (nmol/μL) were determined against a standard concentration curve. Iron (III) content can be calculated as: Iron (III) = Total Iron (II + III) − Iron (II).

### Count and viability assay

After 48 h exposure to ELT [10 μM], DFX [10 μM] and Cytarabine [5 μM], alone and in combination, a cytofluorimetric assay has been performed on the Muse cell analyzer machine using the “Muse^®^ Count and Viability Kit” according to the manufacturer’s instructions. The Muse^®^ Count & Viability Reagent differentially stains viable and non-viable cells based on their permeability to the two DNA binding dyes present in the reagent. The two parameters considered by this assay are viability and nucleated cells. Briefly, 50 μL of a cell suspension (1 × 10^5^ cell/mL) was mixed with 450 μL of Muse^®^ Count & Viability Reagent and incubated for 5 minutes at room temperature in the dark. The results, automatically displayed, were analyzed with “Muse 1.4 Analysis” software for data acquisition and analysis.

### Cell dead and annexin V assay

Apoptosis has been evaluated by a cytofluorimetric assay on the Muse cell analyzer machine using the “Cell dead and Annexin V Assay Kit” according to the manufacturer’s instructions. Test was performed after 48 h of exposure to ELT [10 μM], DFX [10 μM] and Cytarabine [5 μM], alone and in combination. The Muse™ Annexin V & Dead Cell Assay utilizes Annexin V to detect phosphatidylserine (PS) on the external membrane of apoptotic cells. A dead cell marker is also used as an indicator of cell membrane structural integrity, 7-amino-actinomycin D (7-AAD). Briefly, 100 μL of a cell suspension (1 × 10^4^ cells/mL) was mixed with 100 μL of Muse™ Annexin V & Dead Cell Reagent and incubated for 20 min. at room temperature in dark. The results, automatically displayed, were analyzed with “Muse 1.4 Analysis” software for data acquisition.

### Cell cycle analysis

Cell cycle progression was evaluated after 48 h exposure to ELT [10 μM], DFX [10 μM] and Cytarabine [5 μM], alone and in combination, by a cytofluorimetric assay on the Muse cell analyzer machine with the “Cell Cycle Assay Kit” according to the manufacturer’s instructions. The Muse™ Cell Cycle Assay uses a propidium iodide (PI) staining of DNA content to discriminate and measure the percentage of cells in each cell cycle phase (G0/G1, S, and G2/M). The two parameters considered by this assay are cell size and DNA content. Briefly, THP-1 cells were fixed in 70% ice-cold ethanol at 4°C over-night, washed and incubated with Cell Cycle reagent for 30 minutes at room temperature in the dark. The results, automatically displayed, were analyzed with “Muse 1.4 Analysis” software for data acquisition.

### Statistical analysis

Statistical analyses on molecular, biochemical and cellular data were performed using the Student’s *t* test (XLSTAT by Addinsoft 2020, Boston, MA, USA) to evaluate differences between quantitative variables. Data are expressed as mean ± SD and all the experiments were run in technical triplicate. A *p* value ≤ 0.05 (^*^) was considered statistically significant.
